# Building peptidoglycan inside eukaryotic cells: A view from symbiotic and pathogenic bacteria

**DOI:** 10.1111/mmi.14452

**Published:** 2020-03-17

**Authors:** Francisco García‐del Portillo

**Affiliations:** ^1^ Laboratory of Intracellular Bacterial Pathogens National Center for Biotechnology (CNB)‐CSIC Madrid Spain

**Keywords:** endosymbiont, intracellular bacteria, pathogen, peptidoglycan, regulation

## Abstract

The peptidoglycan (PG), as the exoskeleton of most prokaryotes, maintains a defined shape and ensures cell integrity against the high internal turgor pressure. These important roles have attracted researchers to target PG metabolism in order to control bacterial infections. Most studies, however, have been performed in bacteria grown under laboratory conditions, leading to only a partial view on how the PG is synthetized in natural environments. As a case in point, PG metabolism and its regulation remain poorly understood in symbiotic and pathogenic bacteria living inside eukaryotic cells. This review focuses on the PG metabolism of intracellular bacteria, emphasizing the necessity of more in vivo studies involving the analysis of enzymes produced in the intracellular niche and the isolation of PG from bacteria residing within eukaryotic cells. The review also points to persistent infections caused by some intracellular bacterial pathogens and the extent at which the PG could contribute to establish such physiological state. Based on recent evidences, I speculate on the idea that certain structural features of the PG may facilitate attenuation of intracellular growth. Lastly, I discuss recent findings in endosymbionts supporting a cooperation between host and bacterial enzymes to assemble a mature PG.

## Graphical abstract

The peptidoglycan (PG), as main component of the bacterial cell wall, has attracted for decades researchers interested in basic aspects of microbial physiology and the control of microbial infections. Despite the knowledge of PG metabolism gained from bacteria grown under laboratory conditions, the structure and enzymology of the PG in bacteria residing within eukaryotic cells remain poorly characterized. This review compiles the available information about PG metabolism in intracellular bacteria.

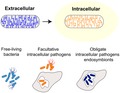

## 1 INTRODUCTION

The peptidoglycan (PG) is a covalently closed macromolecule that defines bacterial shape and preserves cell integrity by withstanding the high internal osmotic (turgor) pressure. The basic PG structure conserved among all eubacteria consists in a glycan backbone made of repeating N‐acetylglucosamine (NAG) and N‐acetylmuramic acid (NAM) units linked by β‐1,4‐glycosidic bonds and cross‐linked by short stem peptides (Pazos & Peters, 2019; Typas, Banzhaf, Gross, & Vollmer, 2012; Vollmer, Blanot, & Pedro, 2008). The peptides attached to NAM are pentapeptides with the sequence l‐alanine (l‐Ala)‐d‐glutamic acid (d‐Glu)‐dibasic amino acid‐d‐alanine (d‐Ala)‐d‐Ala. The dibasic amino acid is normally *meso*‐diaminopimelic acid (*m‐*Dap) or l‐lysine (l‐Lys) in most diderm (Gram‐negative) and monoderm (Gram‐positive) bacteria, respectively. In some Gram‐positive bacteria the stem peptides are linked by additional amino acids that form an interpeptide cross‐bridge. Variations in PG structure were used decades ago for taxonomical purposes (Schleifer & Kandler, 1972).

## 2 THE PG ENZYMATIC MACHINERY

**Figure 1:**
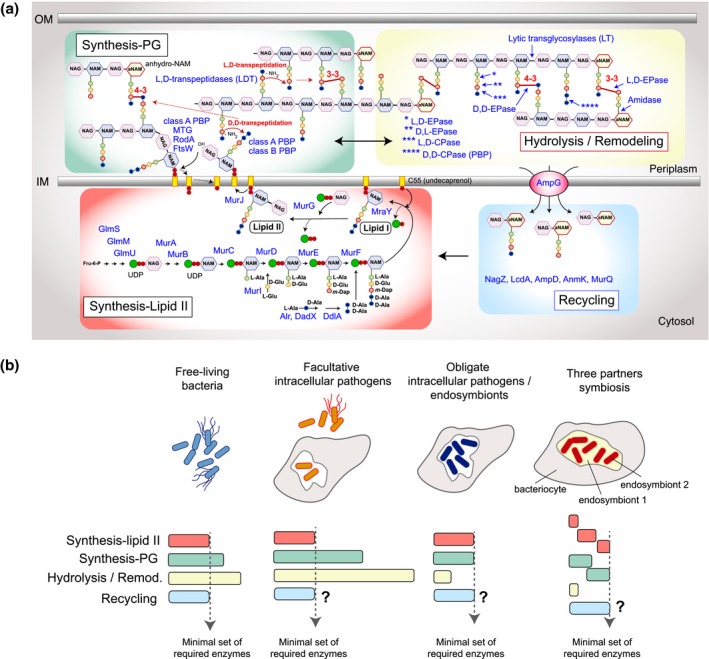
Metabolic routes encompassing the synthesis, hydrolysis, remodeling and recycling of PG and their representation in free‐living and intracellular bacteria. (a) Scheme depicting the cytosolic pathway for synthesis of the precursor unit (lipid II, red); extracytosolic activities involving incorporation of new material by glycosyltransferase and d,d‐ and l,d‐transpeptidation reactions (synthesis of PG, green); reactions of hydrolysis and stem peptide trimming (hydrolysis/remodeling of PG, yellow) and the recycling pathway (recycling of PG, blue). Double headed arrow in the periplasm indicates spatial interconnection between synthesis and hydrolysis/remodeling reactions within the PG meshwork. Names of individual enzymes are indicated for the precursor and recycling routes. The SEDS proteins with glycosyltransferase activity, RodA and FtsW, are also shown. The rest of enzymatic activities and protein families are indicated by generic names: EPase, endopeptidase; CPase, carboxypeptidase; PBP, penicillin‐binding protein. Other abbreviations: NAM, N‐acetylmuramic acid; aNAM, anhydro‐N‐acetylmuramic acid; NAG, N‐acetylglucosamine; OM, outer membrane; IM, inner membrane. (b) Representation of enzymes involved in synthesis of lipid II, PG synthesis, hydrolysis/remodeling and recycling in distinct bacterial types. To date, the recycling route remains poorly characterized in intracellular bacteria. In the case of three‐partner symbiosis, as that of *Candidatus Moranella* (endosymbiont 2) living inside *Candidatus Tremblaya* (endosymbiont 1), this latter living inside bacteriocytes of mealybugs; some enzymes of precursor synthesis are predicted to be provided by genes from the three partners (see Bublitz et al., 2019). Note that many of the periplasmic (extracytosolic) activities are carried out by multiple enzymes

To build the PG sacculus, bacteria synthetize in the cytosol the precursor unit, the lipid II, which is subsequently translocated to the outer leaflet of the plasma membrane (Figure 1a). The enzymes involved in these steps have been extensively characterized in the Gram‐negative *Escherichia coli* (Pazos & Peters, 2019; Typas et al., 2012) and in Gram‐positive bacteria like *Bacillus subtilis* (Bhavsar & Brown, 2006) and *Staphylococcus aureus* (Reed et al., 2015). Synthesis of lipid II requires the formation of UDP‐NAG from fructose‐6‐P, which is transformed to UDP‐NAM‐pentapeptide by the enzymes MurA and MurB and a group of ligases ‐MurC, MurD, MurE, MurF‐, which incorporate amino acids sequentially to the peptide side chain. Key enzymes that fuel this pathway are l‐Glu and l‐Ala racemases (MurI, Alr/DadX), which provide D‐enantiomers to MurD (d‐Glu incorporation) and Ddl, a d‐Ala‐d‐Ala ligase, respectively (Figure 1a). MraY transfers phospho‐NAM‐pentapeptide from UDP‐NAM‐pentapeptide onto the carrier lipid undecaprenol phosphate (C_55_‐P). The resulting molecule, lipid I, is substrate of MurG, which incorporates NAG to generate the lipid II precursor (Typas et al., 2012) (Figure 1a). Lipid II is further flipped to the outer leaflet of the membrane by MurJ (Meeske et al., 2015; Sham et al., 2014) and possibly FtsW (Mohammadi et al., 2011). In some Gram‐positive bacteria like *S. aureus*, *Enterococcus faecalis*, *Streptococcus pneumoniae* and *E. faecium*, dedicated non‐ribosomal peptidyl transferases incorporate additional amino acids to the dibasic amino acid of the stem peptide leading to the formation of interpeptide bridges (Munch & Sahl, 2015; Schneider et al., 2004).

In the extracytosolic (periplasmic) space, the NAG‐NAM‐peptide portion of lipid II is incorporated into the nascent PG by bifunctional (class A) penicillin‐binding proteins (PBPs) harboring glycosyltransferase (GT) and transpeptidase (TP) activities or by monofunctional (class B) PBPs that catalyze TP reactions (Sauvage, Kerff, Terrak, Ayala, & Charlier, 2008; Zapun, Contreras‐Martel, & Vernet, 2008) (Figure 1a). Additional glycosyltransferases contribute to build new PG co‐operating with the morphogenetic class B PBPs. Due to their role in critical events of the bacterial cell cycle, these enzymes are grouped in a protein family known as SEDS, for shape‐elongation‐division‐sporulation (Cho et al., 2016; Meeske et al., 2016). In *E. coli*, the SEDS proteins RodA and FtsW are glycosyltransferases that interact with the monofunctional PBP2 and PBP3, involved in cell elongation and division, respectively (Emami et al., 2017; Meeske et al., 2016; Taguchi, Welsh, et al., 2019). In *E. coli* and *S. aureus*, non‐essential monofunctional polymerases with GT activity also contribute to PG synthesis (Derouaux et al., 2008; Reed, Veiga, Jorge, Terrak, & Pinho, 2011).

Macromolecular PG is constantly remodeled by a large group of periplasmic hydrolases encompassing glycosidases, amidases and peptidases (van Heijenoort, 2011; Vermassen et al., 2019; Vollmer, Joris, Charlier, & Foster, 2008b) and a group of penicillin‐insensitive l,d‐transpeptidases (Hugonnet et al., 2016; Tolufashe et al., 2018). Figure 1a recapitulates the activities of the diverse groups of extracytosolic PG hydrolases. The biosynthetic l,d‐transpeptidases are discussed below specifically in relation to growth rate control.

Other important enzymes are those involved in PG recycling (Johnson, Fisher, & Mobashery, 2013; Park & Uehara, 2008; Reith & Mayer, 2011). It is estimated that, on an average, 30%–50% of the PG in Gram‐negative bacteria is released from the sacculus every generation and most of this material is recycled. The enzymes involved include inner membrane transporters (AmpG) and cytosolic enzymes that allow incorporation of this material to the cytosolic pathway involved in lipid II biosynthesis (Figure 1a). PG recycling has been intensively studied in the physiology of resting, non‐growing bacteria (Uehara & Park, 2008) and as a strategy exploited by bacterial pathogens to minimize exposure to immune defenses (Boudreau, Fisher, & Mobashery, 2012). Turnover products can however accumulate as a result of the uncontrolled activity of lytic transglycosylases that follows antibiotic‐mediated inhibition of PG metabolism. In *Pseudomonas aeruginosa* and some *Enterobacteriaceae* like *Citrobacter freundii* and *Enterobacter cloacae*, the entry of high amounts of anhydro‐muropeptides by the recycling pathway leads to induction of the regulator AmpR and, as consequence, the induction of the chromosomal beta‐lactamase AmpC (Fisher & Mobashery, 2014; Jacobs, Frere, & Normark, 1997). PG recycling has been recently reported in the intracellular pathogen *Mycobacterium tuberculosis* and postulated to interfere with innate immunity as it minimizes the release of stimulatory PG fragments to the external milieu (Moynihan et al., 2019).

## 3 CAN PG STRUCTURE AND ENZYMOLOGY BE MONITORED IN INTRACELLULAR BACTERIA?

Most studies focused on the structure and enzymology of PG have been performed in bacteria grown in the laboratory. Traditionally, this procedure has facilitated the collection of enough PG material for muropeptide separation by high performance liquid chromatography (HPLC), a technique requiring ~200 µg of PG per sample (Alvarez, Hernandez, Pedro, & Cava, 2016; Glauner, 1988; Glauner, Holtje, & Schwarz, 1988). PG is purified from either whole cells or envelope material after boiling in an SDS‐containing solution, with subsequent enzymatic digestions that split the NAM‐β(1‐4)‐NAG glycosidic bond and remove associated proteins and polysaccharides (Desmarais, Pedro, Cava, & Huang, 2013). Unfortunately, these methods involve many ultra‐centrifugation steps that decrease final yields. Current ultra‐sensitive and rapid high‐resolution methods based on ultra‐performance liquid chromatography (UPLC) allow to resolve complex mixtures of more than 50 distinct muropeptide species in 10–20 min (Alvarez et al., 2016). Moreover, novel chromatographic methods based on organic solvents allow in‐line mass spectrometry (MS) of the resolved muropeptides, which was not previously possible in the traditional inorganic method using phosphate buffer in the mobile phase (Alvarez et al., 2016; Glauner, 1988; Glauner et al., 1988). The power of these technological advances is enormous, reflected in studies focused on the analysis of PG chemical diversity in large number of bacterial genera (Espaillat et al., 2016). Despite these technological improvements, PG purification requires a minimal number of bacteria, in the order of 10^10^ cells (Alvarez et al., 2016). This, therefore, continues to be the major obstacle when attempting to purify PG from a reduced number of bacteria, as it is the case in most in vitro and in vivo infection models with intracellular bacterial pathogens and endosymbionts. The few successful cases of muropeptide characterization include those of the obligate bacterial pathogens *Chlamydia trachomatis* (Packiam, Weinrick, Jacobs, & Maurelli, 2015), *Coxiella burnetii* (Sandoz et al., 2016), and *Mycobacterium leprae* (Mahapatra, Crick, McNeil, & Brennan, 2008); the facultative intracellular pathogen *Salmonella enterica* serovar Typhimurium (Quintela, Pedro, Zollner, Allmaier, & Garcia‐del Portillo, 1997); and the *Acanthamoeba* spp. endosymbiont *Protochlamydia amoebophila* (Pilhofer et al., 2013). Attempts to isolate and visualize ‘PG sacculi’ have failed in other cases like that of the deeply rooting chlamydiae *Simkania negevensis* (Pilhofer et al., 2013) or in pathogenic *Chlamydia*. In the latter case, a mid‐cell ring‐link structure was visualized in bacteria using fluorescent D‐amino acid probes that are incorporated specifically in the PG, but no sacculi‐like structure could be purified (Liechti et al., 2016). In the study of Packiam et al. in *C. trachomatis*‐infected epithelial cells, the authors proved the presence of muramyl‐dipeptide (NAM‐l‐Ala‐d‐Glu) and muramyl‐tripeptide (NAM‐l‐Ala‐l‐Glu‐*m*Dap) that stimulated NF‐κB in a NOD2‐dependent manner (Packiam et al., 2015). This study also showed two important facts. First, the characterization by mass spectrometry of additional muropeptides of high mass that supported GT and TP reactions, therefore inferring the existence of PG containing a higher structural organization. Second, the presence of glycine instead of l‐Ala in first position of the stem peptide in some of the characterized muropeptides (Packiam et al., 2015). Overall, these findings categorically resolved the largely discussed ‘Chlamydial anomaly’. Thus, Chlamydiales were known as the only eubacteria that, despite no measurable PG sacculi obtained from infected cells, harbor genes encoding enzymes in PG metabolism and display susceptibility to beta‐lactam antibiotics when located in the intracellular eukaryotic niche (Moulder, 1993).

A similar problem to that encountered when attempting PG structural studies in intracellular bacteria applies for bacterial enzymes that metabolize the PG inside eukaryotic cells. Apart from the detection of PBPs and hydrolases in intracellular persistent *S.* Typhimurium (Castanheira et al., 2017; Rico‐Perez et al., 2016) and a PG amidase in *Rickettsia conorii* and *R. rickettsii* isolated from infected cultured endothelial cells (Patel et al., 2020), there are no reports on the levels of PG enzymes produced by symbiotic and/or pathogenic bacteria located inside eukaryotic cells. Other authors declared no detection of *C. trachomatis* PBPs in infected cell lysates despite good reactivity of the antibodies used and the detection of these enzymes in purified elementary (EB) and reticulate (RB) bodies (Ouellette, Karimova, Subtil, & Ladant, 2012). These observations raise some doubts regarding the protocols used for the recovery of intracellular bacteria from infected cultured cells, which should be contrasted among different laboratories and uniformed as much as possible.

## 4 REDUNDANCY AND SPECIALIZATION OF PG ENZYMES IN INTRACELLULAR BACTERIA

The PG is a dynamic molecule that is constantly hydrolyzed, remodeled and recycled in both actively‐growing and resting bacteria (Cava & de Pedro, 2014; Mueller, Egan, Breukink, Vollmer, & Levin, 2019). A vast number of biosynthetic and hydrolytic extracytosolic enzymes contribute to this PG structural plasticity. On an average, it is estimated that a minimum of 40 extracytosolic enzymes act on different bonds of the PG structure (Chodisetti & Reddy, 2019; Otten, Brilli, Vollmer, Viollier, & Salje, 2018; Sanders & Pavelka, 2013; Scheurwater, Reid, & Clarke, 2008; Vermassen et al., 2019). The bases for so many extracytosolic enzymes that exceed in number to what is needed to synthetize or cleave the bonds existing in the PG (Figure 1b), remain poorly understood. A few of these enzymes are essential, including the class B PBPs involved in cell elongation and division; some class A PBPs, as PBP2 in *S. aureus* (Pinho, Filipe, Lencastre, & Tomasz, 2001); and, hydrolases that participate in cell division (Uehara & Bernhardt, 2011). By contrast, most of the cytosolic reactions required for the synthesis and flip of the lipid II precursor are catalyzed by unique essential enzymes. The exceptions are l‐Ala and L‐Glu racemases, which are encoded by more than one gene in most Gram‐negative and Gram‐positive bacteria (Kang et al., 2011; Oh, Richter, Missiakas, & Schneewind, 2015; Radkov & Moe, 2013).

**Figure 2:**
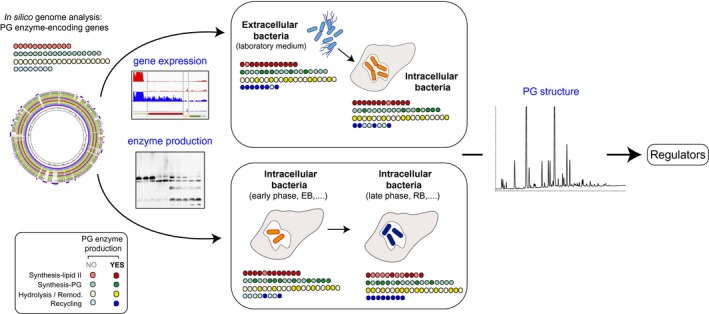
Workflow suitable to increase our still limited knowledge of PG metabolism in intracellular bacteria. The proposed analysis starts with an in silico analysis in the genome of the microorganism of interest to identify genes encoding predicted enzymes involved in PG metabolism. This information is further integrated with gene expression (RNAseq) and protein (proteomics, antibody‐based immunodetection) data. Despite studies reporting gene expression and proteomic data in intracellular bacteria (Jean Beltran, Federspiel, Sheng, & Cristea, 2017), no study has yet determined comprehensively in intracellular bacteria the abundance of the enzymes involved in synthesis, hydrolysis, modification and recycling of PG. Alterations in the levels of defined enzymes are expected to result in structural changes along the intracellular infection. The determination of these alterations can provide valuable clues about the involved regulators, a phenomenon still unexplored for the PG in intracellular bacteria. PG metabolism should be readjusted by facultative intracellular pathogens when they invade host cells (upper panel). In addition, changes are also expected at different post‐infection times or development stages ‐case of elementary body (EB) to reticulate body (RB) transition in Chlamydiae‐ (lower panel)

To what extent enzyme redundancy in PG metabolism occurs in free‐living and intracellular bacteria? Is it possible to see differences among endosymbionts and facultative/obligate intracellular bacteria? To answer these questions, it is necessary to determine which enzymes are produced by bacteria in different niches and to analyze fitness of mutants lacking those enzymes under the same experimental conditions (Figure 2). Early studies supported the idea that many PG enzymes could have redundant functions since they were dispensable with no obvious deleterious effects. Thus, *E. coli* can grow in the absence of all known d,d‐carboxypeptidases (Denome, Elf, Henderson, Nelson, & Young, 1999). The three known l,d‐transpeptidases involved in lipoprotein anchoring to the PG, namely YbiS, YcfS and ErfK, are also dispensable in *S.* Typhimurium (Hernandez et al., 2015). Recent data however demonstrate functional specialization of some enzymes involved in PG metabolism, which perform optimal activities only in defined niches. An example is the d,d‐carboxypeptidase PBP6b of *E. coli*. Unlike other d,d‐carboxypeptidases like PBP4, PBP5, PBP6 and PBP7; PBP6b is the only capable of suppressing a cell shape defect at acidic pH (Peters et al., 2016). Another recent study involving complementation of an *E. coli* PBP5‐null mutant with other d,d‐carboxypeptidases reported divergence in function, concluding that the balanced activity of these d,d‐carboxypeptidases is required to ensure synthesis of a robust mature PG (Meiresonne, Ploeg, Hink, & Blaauwen, 2017). Similar cases of specialization include the bifunctional enzymes PBP1a and PBP1b of *E. coli*, with display optimal GT and TP activities at alkaline and acidic pH, respectively (Mueller et al., 2019). Singh et al. also identified in *E. coli* three periplasmic PG endopeptidases, Spr, YdhO and YebA, all dispensable in laboratory media but with the requirement of at least one of the three for growth (Singh, SaiSree, Amrutha, & Reddy, 2012). *S.* Typhimurium up‐regulates a specialized PBP that is active only in acidic pH and promotes cell division in intracellular bacteria (Castanheira et al., 2017). This work also showed the capacity of this ‘intracellularly‐induced PBP’ to drive cell division in mutants lacking PBP3, which is essential in *E. coli* (Castanheira, Cestero, Garcia‐del Portillo, & Pucciarelli, 2018; Castanheira et al., 2017). These latter findings highlight the suitability of working with facultative intracellular bacterial pathogens to uncover new phenomena related to PG plasticity and specialization of PG enzymes (Figure 2). *M. tuberculosis* also illustrates other examples of enzyme specialization. The genome of this pathogen encodes five resuscitation‐promoting factors (Rpf) paralogs that show homology with lysozyme and lytic transglycosylases and that allow latent bacteria to resume growth (Rosser, Stover, Pareek, & Mukamolova, 2017). Lack of the membrane‐bound RpfA and RpfB is sufficient to observe defects in reactivation from chronic tuberculosis and innate immunity evasion (Russell‐Goldman, Xu, Wang, Chan, & Tufariello, 2008). *M. tuberculosis* has also five l,d‐transpeptidases (Squeglia, Ruggiero, & Berisio, 2018). The loss of only LdtMt5 leads to aberrant growth and increased antibiotic susceptibility (Brammer Basta et al., 2015). Moreover, while the *M. tuberculosis* genome encodes two glycosyltransferases and four transpeptidases, the absence of the glycosyltransferase RodA and the transpeptidase PbpA affects pathogen survival in granulomas using a guinea pig infection model despite no effect in vitro for growth in the laboratory media (Arora, Chawla, Malakar, Singh, & Nandicoori, 2018). In another study, Reed et al. succeeded in deleting in *S. aureus* seven of the nine genes encoding enzymes bearing GT and TP domains (Reed et al., 2015). The mutant carrying the minimal PG biosynthesis machinery does not show a growth phenotype in laboratory media, however it is impaired for virulence and become highly susceptible to antibiotics (Reed et al., 2015). Another case of enzyme specialization includes the lytic transglycosylase RlpA, which is absolutely required for separation of *Vibrio cholerae* cells in low salt medium (Weaver et al., 2019). A lesson to learn from these studies is that restricting the study of PG metabolism to laboratory conditions can hide the action of defined enzymes or structural variations in the PG that might be relevant in other, more natural niches. This fact alerts us about the necessity of increasing the number of in vivo studies, relatively scarce to date.

## 5 PG STRUCTURAL VARIATIONS AND THE CONTROL OF INTRACELLULAR GROWTH RATE

Modifications in the basic PG structure are of major interest. Most of them involve changes in defined sites of the NAM or NAG sugars or the amino acids of the side chain (Pazos & Peters, 2019). In some instances, there is also incorporation of non‐canonical amino acids (Cava, Pedro, Lam, Davis, & Waldor, 2011). These changes denote the plasticity in PG structure and distinct modes of PG synthesis in bacteria occupying diverse habitats (Turner, Vollmer, & Foster, 2014; Zhang, Lin, Xing, & Zhang, 2018). Alterations in PG may have also evolved from the selective pressure imposed by innate immune host defenses (Brott & Clarke, 2019; Wolf & Underhill, 2018); or, as it occurs in some antibiotic‐producing actinomycetes, by the synthesis of molecules that target PG metabolism (Unsleber, Wohlleben, & Stegmann, 2019). This mini‐review does not intend to recapitulate current knowledge on naturally‐occurring PG modifications and immune evasion, recommending to the reader excellent recent reviews addressing these topics (Brott & Clarke, 2019; Callewaert et al., 2012; Cava & de Pedro, 2014; Dziarski & Gupta, 2018; Neyen & Lemaitre, 2016; Wheeler, Chevalier, Eberl, & Gomperts Boneca, 2014; Wolf & Underhill, 2018).

Instead, I would like to discuss alterations in PG structure that are controlled ‘within the host’ when bacterial pathogens colonize the intracellular eukaryotic niche (Pucciarelli & Garcia‐del Portillo, 2018). If common variations in PG structure are found in facultative intracellular bacterial pathogens, obligate intracellular pathogens and endosymbionts, one could assume such changes may facilitate immune evasion. The intracellular receptors NOD1 and NOD2 evolved to recognize distinct molecular patterns in the PG and are by far the main host factors that influence the fate of intracellular infections (Shaw, Reimer, Kim, & Nunez, 2008). However, the picture is still fragmented and the changes that might be triggered only upon sensing host cues remain poorly understood. An illustrative case is that of *M. tuberculosis*, that synthetizes an altered PG with a percentage of their d‐Glu and *m*‐Dap residues amidated as well as a fraction of NAM residues with a glycol‐derivation instead of acetylation (Angala, Belardinelli, Huc‐Claustre, Wheat, & Jackson, 2014; Squeglia et al., 2018). These modifications have been shown to impair NOD2 signaling using synthetic PG fragments (Wang et al., 2016). Whether these changes are predominant in the PG of intracellular *M. tuberculosis,* remains however to be demonstrated. Noteworthy, the PG of intracellular *M. leprae* is substituted with glycine instead of l‐Ala as first residue of the side chain (Draper, Kandler, & Darbre, 1987; Mahapatra et al., 2008) with no apparent interference with NOD2 signaling (Schenk et al., 2016). As above mentioned, substitution of l‐Ala by glycine has also been detected in soluble PG fragments obtained from *C. trachomatis*‐infected cells (Packiam et al., 2015). If we believe this modification means no alteration of NOD2‐signalling, one should conclude that obligate intracellular pathogens may require some level of stimulation of intracellular defenses to control growth. This concept of delicate balance between stimulation and evasion of immune defenses has been discussed by others (Mitchell & Isberg, 2017).

Is there any evidence supporting strategies by intracellular bacterial pathogens or endosymbionts based on alteration of PG structure in order to stimulate innate defenses or to self‐attenuate growth? We certainly need additional structural studies involving PG extraction from intracellular bacteria in distinct physiological conditions (low vs. high intracellular growth rate, i.e. persistence vs. proliferation) and intracellular parasitism versus symbiosis. Growth attenuation is a hallmark of intracellular bacteria that establish an intracellular persistent infection, but we do not know how PG metabolism contributes to this phenomenon. I would like to speculate on a putative role of l,d‐transpeptidases to restrain active growth. These enzymes catalyze the formation of cross‐links between amino acids in third position of adjacent stem peptides, l,d‐(3‐3) bridges of structure *m‐*Dap‐*m‐*Dap (Pidgeon et al., 2019). Unlike the d,d‐(4‐3) bridge catalyzed by PBPs, the l,d‐(3‐3) bridge does not depend on an intact pentapeptide side chain and, as consequence, can be formed in structured mature PG (Figure 1a). *E. coli* mutants defective in multiple l,d‐transpeptidases are viable in the laboratory (Sanders & Pavelka, 2013). In *M. tuberculosis*, in which the fraction of l,d (3‐3)‐bridges can reach up to 80% of the total in stationary phase (Lavollay et al., 2008), the genetic inactivation of l,d‐transpeptidases or their inhibition by carbapenems result in aberrant shape and growth defects (Brammer Basta et al., 2015; Maitra et al., 2019). Inhibition of l,d‐transpeptidases by carbepenems is currently an intense area of study in drug therapy against tuberculosis (Gokulan & Varughese, 2019; Squeglia et al., 2018). It is worth to note that the l,d (3‐3)‐bridge is prominent in the PG of the obligate intracellular pathogen *C. burnetii* (Sandoz et al., 2016). Although in extracellular pathogens like *Clostridium difficile* the l,d (3‐3)‐bridge is also abundant, it represents a minor fraction (<5% of total cross‐links) of other free‐living bacteria like *E. coli* during active growth while it increases about two‐fold in stationary phase (Pisabarro, Pedro, & Vazquez, 1985). The l,d‐(3‐3)‐bridge confers beta‐lactam resistance since it is formed upon cleavage of a diamino‐acid‐d‐Ala bond and it has been shown in some bacteria to withstand outer membrane damage (More et al., 2019). However, its increase when free‐living bacteria arrest growth and its prevalence in some intracellular bacteria may indicate that it might be evolved to impair expansion of the PG sacculus. With this idea in mind, I assign to the l,d‐(3‐3)‐bridge a role as ‘corset’ leading to an intentional reduction of the growth rate. As consequence, this cross‐linking could facilitate the establishment of a persistence stage inside eukaryotic cells. In which evidence(s) do I stand for these statements? Facultative intracellular bacterial pathogens transit constantly from extracellular nutrient‐rich conditions to an intracellular lifestyle accompanied, in most instances, by a reduction in growth rate. Intriguingly, data that we collected on PG structure of intracellular *S. enterica* serovar Typhimurium proliferating inside HeLa cells failed to show major increase in l,d (3‐3)‐bridges (Quintela et al., 1997). However, our more recent data obtained in fibroblasts persistently infected with this pathogen demonstrate that non‐growing intracellular bacteria increase notoriously the percentage of l,d (3‐3)‐bridges compared to the stationary phase bacteria used to infect the cells (García‐del Portillo, unpublished). Therefore, intracellular host cues associated with a reduction in growth rate may ‘stimulate’ the formation of l,d (3‐3)‐bridges. Moreover, our recent observations in *S.* Typhimurium mutants lacking l,d‐transpeptidases show that the absence of l,d (3‐3)‐bridges result in exacerbated growth in a medium that mimics the phagosomal environment with rates higher than the displayed by wild type bacteria (García‐del Portillo, unpublished). Consistent with these findings, l,d‐transpeptidation is down‐regulated by *S.* Typhimurium to resist exposure to bile salts, a phenomenon that occurs extracellularly (Hernandez et al., 2015).

In this line of reasoning, we should however not discard a broader physiological role for the l,d (3‐3)‐bridge. l,d‐transpeptidases are not exclusive of slow‐growing bacteria or intracellular bacterial pathogens and, as above mentioned, are also present in free‐living bacteria and fast growing mycobacteria like *M.* *smegmatis* (Zandi, Marshburn, Stateler, & Brammer Basta, 2019). In Gram‐negative bacteria, l,d‐transpeptidation also play a housekeeping role in the covalent anchoring of the outer membrane Braun lipoprotein to the PG (Magnet et al., 2007). l,d (3‐3)‐bridges are also recognized by dedicated endopeptidases (Chodisetti & Reddy, 2019; Vollmer & Bertsche, 2008) and the lack of some of these hydrolases result in growth defects that are abrogated in the absence of l,d‐transpeptidases (Chodisetti & Reddy, 2019). Such loss of cleavage in the l,d (3‐3)‐bridges could compromise the transit of facultative intracellular pathogens between host and non‐host environments, a tempting hypothesis to explore in future studies.

In summary, the physiological role of the l,d (3‐3) bridge needs to be further investigated despite its low abundance as a reduced fraction of PG cross‐links. Recent studies have proved that intracellular *S.* Typhi exploits a l,d‐transpeptidase to modify PG structure allowing typhoid secretion (Geiger, Pazos, Lara‐Tejero, Vollmer, & Galan, 2018) and that l,d‐transpeptidases assemble an unique PG that withstand defects in outer membrane assembly (More et al., 2019). This latter finding fits with the speculation of a PG enriched in l,d (3‐3) bridges that has been strengthened to act as a ‘security corset’, probably attenuating growth, in those cases in which outer membrane integrity is compromised. Some intracellular bacterial pathogens shed large amounts of outer membrane material inside the eukaryotic cells (Garcia‐del Portillo, Stein, & Finlay, 1997) and they depend on the extra‐cytoplasmic stress‐responding sigma factor RpoE to survive inside the host cell (Cano et al., 2001). Consistently, RpoE stabilizes the membrane against host‐damaging agents such as antimicrobial peptides or reactive oxygen and nitrogen species (Mitchell & Silhavy, 2019). Future studies should therefore analyze probable functional connections between RpoE and changes in PG structure, perhaps affecting the relative abundance of l,d‐3‐3‐bridges.

## 6 GAIN AND LOSS OF PG ENZYMES IN INTRACELLULAR PATHOGENS: OBLIGATE VERSUS FACULTATIVE

Similar to endosymbiotic bacteria, the genome size of obligate intracellular bacterial pathogens like Chlamydiae or Rickettsiae is relatively small, in the 1–2 Mb range, considerably smaller than those of free‐living or intracellular facultative bacterial pathogens (3–5 Mb). Chlamydiales are Gram‐negative bacteria with a biphasic life cycle that alternates between the extracellular and less metabolically active elementary body (EB) and the intracellular and highly active reticulate body (RB) (Klockner, Buhl, Viollier, & Henrichfreise, 2018; Moulder, 1991). The Chlamydiales genome has lost many of the genes encoding periplasmic enzymes involved in PG remodeling as the d,d‐carboxypeptidases. Only one member of this protein family, a DacC homologue, is identified (Jacquier, Viollier, & Greub, 2015). Many cell division proteins, like the tubulin homologue FtsZ, are also missing in Chlamydiales with a division process that is orchestrated by the actin homologue MreB and its interactors RodZ and the LysM‐domain‐containing protein AmiA (NlpD), this latter with dual amidase and carboxypeptidase activities (Jacquier, Frandi, Viollier, & Greub, 2015; Jacquier, Viollier, et al., 2015; Klockner et al., 2018, 2014). Such reduced number of periplasmic PG hydrolases is a hallmark of obligate intracellular pathogens (Otten et al., 2018) and endosymbionts (Wilmes et al., 2017), contrasting with the more than 35 PG hydrolases known in free‐living bacteria like *E. coli* (van Heijenoort, 2011; Vollmer, Joris, et al., 2008b) (Figure 1b). Regarding proteins involved in PG biosynthesis, Chlamydiales have only two PBPs with TP domains that are homologs to *E. coli* PBP2 and PBP3(FtsI) and no enzyme known with canonical GT activity. Nonetheless, both RodA and FtsW homologues exist in Chlamydiales and these two proteins were recently shown to act as non‐canonical GT assisting incorporation of lipid II precursor into the PG with their partners PBP2 and PBP3, respectively (Emami et al., 2017; Meeske et al., 2016; Taguchi, Welsh, et al., 2019).

Another interesting feature of Chlamydiales is the conservation of the entire pathway of lipid II except for the Glu racemase (MurI) and with GlyA proposed to be the Ala racemase (De Benedetti et al., 2014; Jacquier, Viollier, et al., 2015). Until recently, there was no formal evidence of the presence of PG in intracellular Chlamydiae despite their susceptibility for beta‐lactams, becoming the presence of an intact lipid II route enigmatic (Henrichfreise et al., 2009). The recent discovery of PG fragments *Chlamydia*‐infected cells and other lineages of *Chlamydia*‐related bacteria like *Protochlamydia* and *Waddlia* (Klockner et al., 2018) supports the conservation of the cytosolic pathway responsible for lipid II biosynthesis.

In contrast to Chlamydiales, other obligate intracellular pathogens like bacteria of the genus *Rickettsia* have conserved a larger repertoire of periplasmic PG enzymes, including amidases (Patel et al., 2020) and bifunctional PBPs with GT and TP domains (Otten et al., 2018). The obligate pathogens *C. burnetti* and *M. leprae* have also maintained a large number of genes encoding periplasmic PG enzymes, especially biosynthetic class A and class B PBPs (Otten et al., 2018). The presence of a reasonable number of genes related to PG metabolism correlates with the production of a PG that can be purified and analyzed at the structural level (Mahapatra et al., 2008; Sandoz et al., 2016).

Why extracytosolic ‐periplasmic‐ enzymes acting on the PG are drastically reduced in bacteria adapted to intracellular niches? A possibility is that the PG could behave as sensor device that responds to external insults and transmits the signals to the cytosolic environment. The induction of the chromosomal beta‐lactamase AmpC following antibiotic‐mediated blockage of new PG synthesis is a clear example (Fisher & Mobashery, 2014). If the PG plays such sentinel role, we should expect larger numbers of periplasmic enzymes in extracellular or facultative intracellular pathogens encountering a larger variety of niches than in obligate intracellular pathogens or endosymbionts.

I am particularly intrigued with this idea of environment sensing. A recent study revealed that the genome of the intracellular pathogen *Listeria monocytogenes* encodes three RodA (RodA1, RodA2, RodA3) and two FtsW (FtsW1, FtsW2) paralogs (Rismondo, Halbedel, & Grundling, 2019). Deficiencies in some of these proteins result in changes in shape and antibiotic resistance, therefore displaying capacity for a functional interchange in the laboratory medium. Unfortunately, these mutants were not tested for the intracellular infection, which it could have been valuable to discern whether some of these paralogs evolved for a virulence‐related trait. The entire set of RodA and FtsW paralog‐encoding genes is present in the genome of non‐pathogenic *Listeria* species like *L. innocua*. However, in the absence of studies at protein level, we cannot conclude whether they are equally used by pathogenic and non‐pathogenic *Listeria*.

Regulation of PG synthesis is also instrumental for *S.* Typhimurium to colonize the intracellular niche. Beside the synthesis of a pathogen‐specific PBP in such environment, this pathogen up‐regulates an D,L‐endopeptidase with a NlpC/P60 domain that cleaves the d‐Glu‐*m‐*Dap bond when is located within the phagosome (Rico‐Perez et al., 2016). Such activity could minimize release of PG fragments bearing this motif known as potent NOD1 inducers. Analogous activities decreasing the relative levels of stem peptides bearing immunostimulatory motifs have been described in *Helicobacter pylori* (Costa et al., 1999) and *Campylobacter jejuni* (Frirdich et al., 2019). These two pathogens undergo transition to a coccoid morphology concomitant to an increase in muropeptides with short dipeptide side chains at expenses of those having tri‐ and tetrapeptides. For both pathogens, the coccoid morphology is achieved following action of dedicated amidases and endopeptidases resulting in a PG with reduced capacity to activate NOD1 (Chaput et al., 2006; Frirdich et al., 2019). Intracellular *Legionella pneumophila* also encodes a periplasmic protein, EnhC, which impairs activity of the PG glycosidase Slt to minimize release of immunostimulatory PG fragments (Liu et al., 2012). These findings certainly prove that some bacterial pathogens have increased their arsenal of periplasmic PG enzymes to promote adaptation to the intracellular eukaryotic niche.

## 7 CELL WALL DEFICIENCY IN INTRACELLULAR BACTERIA

Work from decades has accumulated evidence supporting the capacity of many bacteria to switch to a physiological state in which they undergo growth and division in the absence of cell wall (Claessen & Errington, 2019). These cell wall deficient bacteria, known as L‐forms, have been extensively studied in Gram‐positive bacteria like the intracellular pathogen *L. monocytogenes* (Studer, Borisova, et al., 2016a; Studer, Staubli, et al., 2016) and *Bacillus subtilis* (Kawai, Mickiewicz, & Errington, 2018). How this physiological state is represented in the adaptation process to the intracellular lifestyle is currently unknown. Intriguingly, Errington and colleagues have recently shown that *B. subtilis* can enter into the L‐form state when coping stress inside macrophages and that these bacteria retain viability (Kawai et al., 2018). Host PG degrading enzymes, like lysozyme and PGLYRP2, are proposed to induce emergence of these cell wall deficient intracellular bacteria (Kawai et al., 2018). Unlike persistent dormant bacteria, L‐forms grow in the presence of beta‐lactam antibiotics and this is much concern to control bacterial infections. Indeed, L‐forms have been reported in recurrent and chronic bacterial infections (Claessen & Errington, 2019). Whether L‐forms also emerge in intracellular bacteria following infection of non‐phagocytic cells has not been addressed. It would be also of much interest to analyze L‐form emergence in bacteria that modify the sugar backbone of the PG to resist lysozyme attack. Some of these bacteria are intracellular pathogens, like *L. monocytogenes*, and mutants defective in these modifications are known to be attenuated in pathogenicity (Aubry et al., 2011).

## 8 DOES THE HOST PARTICIPATE IN PG METABOLISM OF INTRACELLULAR BACTERIA?

To date, it is not yet clear how obligate intracellular bacterial pathogens ‐most causing long lasting persistent infections‐ or endosymbionts deal with PG biosynthesis and avoid immune recognition while encoding, most of them, a minimal set of enzymes (Figure 1b). Recent studies however provide new insights into these unique bacterial‐host interactions. Some obligate intracellular bacteria like *Wolbachia* spp., which develop an intracellular lifestyle in arthropods or nematodes, have maintained genes encoding PG‐hydrolases (amidases). A recently identified amidase, AmiD, was observed only in species infecting arthropods (Wilmes et al., 2017), suggesting that this hydrolase contributes to avoid immune recognition by degrading cell wall fragments in the periplasm. Interestingly, the genome of *Wolbachia* spp. does not encode biosynthetic glycosyltransferases, augmenting the probability of releasing PG fragments. This fact may explain the inflammation associated to human filariasis, a disease caused by the nematode *Brugia malayi*, which harbors a *Wolbachia* endosymbiont (Foster et al., 2005).

A completely new view has recently put into scene by two studies involving the insect endosymbionts *Buchnera* spp. (Chung, Jing, Luo, & Douglas, 2018) and *Candidatus Moranella*. This latter endosymbiont lives inside another bacterium, *Candidatus Tremblaya*, which also lives inside specialized insect cells ‐bacteriocytes‐ of mealybugs (Bublitz et al., 2019).

In the first study of Chung et al. analyzing the aphid‐*Buchnera* symbiosis, the authors demonstrated that interference of *amiD* and *ldcA1*, two PG‐enzymes encoding genes acquired by horizontal transfer by the pea aphid *Acyrthosiphon pisum*, resulted in reduced bacterial loads (Chung et al., 2018). These authors concluded that these two host enzymes could clean PG products derived from *Buchnera* to minimize in this manner immune recognition. Intriguingly, *amiD* and *ldcA1* are overexpressed in the bacteriocytes (Chung et al., 2018). As some endosymbionts are essential for insect development, controlling endosymbiont fitness by altering expression of host genes encoding PG enzymes is currently intensively investigated to control insect pests.

The second study of Bublitz et al. bring us another new concept: the cooperation of genomes from the three interacting partners, the mealybug *Planococcus citri*, the betaproteobacterium *Candidatus Tremlaya* and the gammaproteobacterium *Candidatus Moranella*, to assemble a competent PG biosynthetic route for lipid II synthesis. This cooperation was manifested by the visualization of a PG sacculus in the *Candidatus Moranella* surface using fluorescent probes (Bublitz et al., 2019). The first enzymes required to lipid II synthesis, GlmS, GlmM, and GlmU, which metabolize fructose 6‐phosphate to UDP‐NAG (Figure 1a), are encoded in the mealybug genome. Their role is complemented by cytosolic enzymes encoded in the *Tremblaya* and *Moranella* genomes, including those involved in lipid II synthesis, its flipping to the periplasm and subsequent incorporation into a mature PG. Interestingly, some of these bacterial enzymes are predicted to be acquired by horizontal transfer from other bacteria (e.g. Bacteroidetes) (Bublitz et al., 2019). The most puzzling aspect of this co‐operation is that the enzymatic activities offered by the insect genome are needed in the *Moranella* cytosol to build the lipid II precursor. How the insect enzymes could reach the cytosol of the *Moranella* cells was, however, not determined.

## 9 FUTURE DIRECTIONS

The interest in the PG has increased enormously during the last decade. A bulk of new data on the biology of this macromolecule has been possible due to novel approaches like those involving in vivo incorporation of fluorescent D‐amino acid derivate probes by endogenous transpeptidases (Hsu, Booher, Egan, Vollmer, & VanNieuwenhze, 2019; Taguchi, Kahne, & Walker, 2019). This allows to monitor in a precise temporal and spatial manner the dynamics of PG metabolism in terms of growth and turnover (Hsu et al., 2019). In addition to the classical biochemical analysis on the structure of PG, there had been major advances in chromatographic and analytical techniques (Alvarez et al., 2016; Espaillat et al., 2016). Likewise, structural studies in different classes of PG have allowed to dissect the molecular bases of natural substrate recognition or drug inhibition (Caveney, Li, & Strynadka, 2018; Tolufashe et al., 2018).

Despite these advances, other areas remain poorly investigated. An example is the ‘regulation of PG enzymology’ for which scarce information exists for basically all bacterial groups. It is rather surprising that there is still not knowledge on a global basis about the repertoire of enzymes involved in PG metabolism that are produced in a defined condition (Figure 2). This is especially relevant for enzymes predicted to act in identical bonds of the PG sacculus ‐the repeatedly discussed redundancy‐ or for those enzymes with multiple paralogs of yet unknown function. The global analysis performed ‘at the protein level’ will be, in my opinion, insightful to understand why these apparent multiple copies exist and if compensatory effects exist. A second level of uncertainty refers to regulators, acting at the transcriptional or post‐transcriptional level, that control expression and activity of PG enzymes and for which we are still at a very early stage of knowledge. Some exceptions include: (a) the essential transmembrane protein kinases bearing PASTA (penicillin‐binding protein and serine/threonine kinase associated) domains that control PG synthesis and cell wall homeostasis in low G + C Gram‐positive bacteria (Dubrac, Bisicchia, Devine, & Msadek, 2008; Jones & Dyson, 2006); (b) the sigma‐type regulators that regulate expression of PG enzymes driving the synthesis of an unique PG during spore formation and germination in the mother cell, the forespore and the mature spore (Popham & Bernhards, 2015); and, (c) proteins that associate in complexes with synthases and hydrolases regulating their activity by protein‐protein interactions (Egan, Cleverley, Peters, Lewis, & Vollmer, 2017; Pazos, Peters, & Vollmer, 2017). Intriguingly, most PG enzymes controlled by these regulators are periplasmic synthases and hydrolases, reinforcing the idea of macromolecular PG as a sensor device that integrates external signals upon exposure to stress. Our current knowledge on regulation leaves still open important questions as how the synthesis of lipid II and incorporation of new muropeptides into the growing PG, is regulated.

When translating regulation of PG metabolism to intracellular bacterial pathogens, there are additional challenges. These challenges include the isolation of sufficient PG and protein material to determine relative levels of enzymes, structural details of the PG and the chemical nature of PG fragments shed by bacteria when they are inside the eukaryotic cell. These are new avenues of investigation that will add relevant information to studies that, up to now, provide only partial or indirect evidence on the action of defined PG enzymes or PG structures. Phenotypic analyses based exclusively on mutants lacking defined PG enzymes are frequently performed but they do not provide a formal evidence of the essentiality of a particular enzyme in a defined niche. Thus, the absence of an enzyme can be compensated by expression of an enzyme with similar activity that is normally ‘not expressed’ in the condition under study. Furthermore, unless PG is purified from intracellular bacteria, it is not possible to definitively conclude the selective pressure that the host has imposed on intracellular pathogens. As my group and a few others have shown (Mahapatra et al., 2008; Packiam et al., 2015; Pilhofer et al., 2013; Quintela et al., 1997; Sandoz et al., 2016), it is possible to isolate PG directly from the infected cells and this information is crucial to understand these host‐pathogen interactions. Another fascinating fact is the presence of PG containing D‐amino acids in moss chloroplasts (Hirano et al., 2016). Interestingly, the moss genome contains genes encoding Mur proteins involved in lipid‐II biosynthesis that, if deleted, result in defects in chloroplast division (Hirano et al., 2016). Antibiotic treatment that block PG synthesis also affect chloroplast division in the algae *Closterium* (Matsumoto, Takechi, Sato, Takio, & Takano, 2012). A complete set of genes involved in PG synthesis is also found in some plants and algae, although most were found not to be of cyanobacterial origin by comparative genomic analyses (Sato & Takano, 2017). Host contribution to PG metabolism of endosymbionts or organelles is an interesting area of research from which we can much learn to understand the interaction of intracellular bacterial pathogens with eukaryotic cells.

## CONFLICT OF INTEREST

The author declares no conflict of interest.
